# A study on the effect of slotted airfoil on the performance of Darrieus vertical axis wind turbines in different wind regions

**DOI:** 10.1371/journal.pone.0334110

**Published:** 2025-10-07

**Authors:** Liqi Luo, Qiuyun Mo, Yuefeng Li, Tao Jiang, Yinglei Zhao

**Affiliations:** School of Mechanical and Electrical Engineering, Guilin University of Electronic Technology, Guilin, Guangxi, China; National University of Sciences and Technology, PAKISTAN

## Abstract

Slotted airfoils mitigate the flow separation on the blades operating at high angles of attack in the upwind region, consequently augmenting the power coefficient and reducing the startup wind speed of Darrieus vertical axis wind turbines (VAWTs). Nonetheless, the presence of the slot structure alters the original flow dynamics, inducing flow separation when the blade operates in the downwind region and at elevated blade tip speed ratios (TSR), which leads to a reduction in the blade’s power coefficient. This study establishes an aerodynamic model of the flow field migration around the blade surface by utilizing the lattice Boltzmann method in conjunction with large eddy simulation to ascertain the influence of the inlet and outlet positions of the slot on the flow field structure across different wind regions. The simulations indicate that, under the downwind region and at high TSR, positioning the slot at the midsection of the blade, although it expands flow separation near the trailing-edge, does not disrupt the primary flow at the leading-edge. Unexpectedly, the slot optimizes the pressure distribution on the pressure side of the blade, thereby enhancing the blade’s performance in the downwind region. At a TSR of 3.3, the average power coefficient of the blades in the downwind region increases by up to 63.62%. These results offer valuable insights for the implementation of slotted airfoils to enhance energy conversion efficiency in VAWTs’ design optimization.

## 1. Introduction

Vertical-axis wind turbines (VAWTs) provide an effective clean energy solution for remote areas far from integrated grid systems and where large wind farms cannot be installed in complex built environments such as cities [1–3]. Currently, Darrieus VAWTs suffer from issues such as high start-up wind speeds, low power coefficients, and low power densities [3,4]. The primary factor contributing to these issues is that the blades generate extensive flow separation at large angles of attack (AOA), leading to the formation of dynamic stall vortices (DSVs), in turn, diminish the efficiency of the VAWT [5,6]. For thick airfoils (e.g., NACA0021), DSVs initially form at the trailing-edge of the airfoil and progressively move towards the leading-edge as the AOA increases, resulting in a sharp decrease in the blade power coefficient [7]. The slotted airfoil technique, which was initially introduced [8,9] to enhance the lift of aircraft wings, is a passive flow control (PFC) method [10] that effectively weakens the flow separation of blades at large AOA and extends the high lift range of the airfoil [11,12].

The parameters of the slots have a direct impact on the performance of the blade. Prior research has demonstrated that while slotted airfoils can substantially enhance the lift force of the blade at high AOA [13,14], at low AOA, the extent of the flow separation zone on the blade is limited. Under these conditions, the interference of the slot jet to the flow structure on the suction side leads to a reduction of lift force and an increase in drag forces [14]. At low TSR, VAWTs exhibited considerable fluctuations in the AOA throughout their rotational cycle. The use of slotted airfoils contributes to mitigating flow separation in the upwind region (0° ≤ *θ* ≤ 180°) of Darrieus VAWTs [15,16]. Nevertheless, as the TSR increases, the range of AOA variations during the rotational cycle diminishes, which tends to reduce flow separation. Under conditions of small AOA, the effectiveness of the slotted airfoil is constrained and may adversely affect performance, resulting in a power coefficient for the slotted airfoil that is lower than that of the baseline airfoil. Moreover, when the slotted airfoil operates in the downwind region(180° ≤ *θ* ≤ 360°), the suction and pressure sides of the blades are reversed owing to wind region switching. This reversal results in the interchange of the slot inlet and outlet, which causes a reversal of the jet flow. The reversed slot jet near the leading-edge disrupts the attached flow structure on the suction side within the downwind region, leading to flow separation and a decrease in the airfoil moment coefficient [16]. Therefore, exploring the impact of various slotted positions on the performance of slotted airfoils across different wind regions and effectively limiting the adverse impacts at high TSR is essential for improving the structural design of slotted airfoils and enhancing energy utilization.

This study analyzes the influence of NACA0021 slotted airfoil operating in different wind regions on the performance of Darrieus VAWT. It aims to identify the optimal parameters for the slotted location to enhance energy utilization. Given the prohibitive computational demands of three-dimensional simulations, this study utilizes 2D numerical modeling via lattice Boltzmann method in conjunction with large eddy simulation (LBM-LES) framework. This methodology facilitates precise modeling of flow separation and DSV structure around the blade. Furthermore, it allows for a detailed analysis of how various inlet and outlet positions on the airfoil affect blade flow structure across different wind regions at TSRs of 0.5, 2.4, and 3.3.

## 2. Numerical methodology and model validation

### 2.1 Numerical methodology

The LBM is a particle-based fully Lagrangian method suitable for meshless technology, and the principal computational domain does not need to be fitted with a mesh during the solution process. The LBM circumvents the mesh distortion issues commonly encountered in traditional finite-element and finite-volume methods, thereby facilitating accurate analysis of complex geometrical boundaries, multiphase flows, small-gap flows, and fluid-structure interactions [17–19]. The method integrates the wall adaptive local eddy viscosity model (WALE) and is applied for the performance prediction of the Darrieus VAWT, which can obtain computational results that are more consistent with the experimental data [20]. The WALE model is designed to simulate LES in turbulent boundary layers within transitional flows [21,22] and is applied to complex turbulence simulations in close agreement with the results of direct numerical simulations (DNS) [23]. Fluid analysis software such as Xflow provides the above-mentioned sophisticated numerical computation techniques.

### 2.2 Geometric modelling

**Fig 1:**
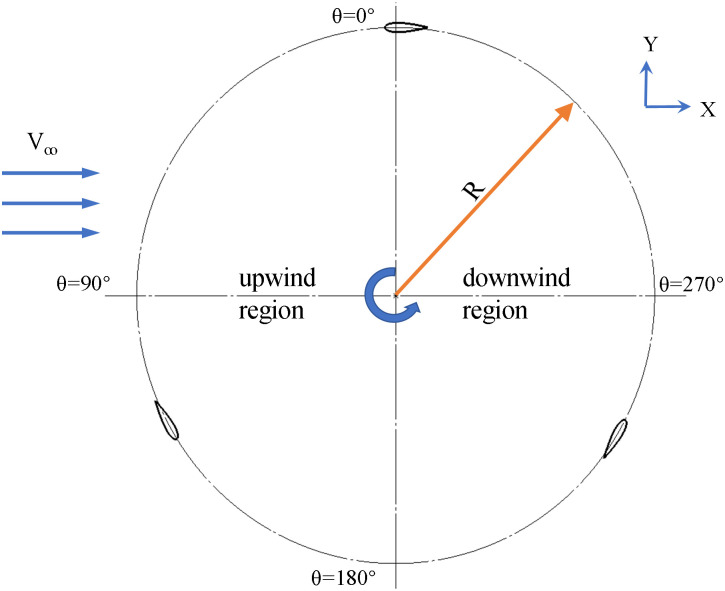
Top view of H-type Darrieus VAWT wind turbine geometry.

In this study, the slotted airfoil design was adopted from the VAWT structure of the straight-bladed (H-type) Darrieus VAWT performance measurement experiment conducted by Battisti et al. [24] in 2018, the structure and parameters of the baseline airfoil are indicated in Table 1 and Fig 1.

**Table 1:** Main geometric parameters of H-type Darrieus VAWT [24].

Airfoil	Blade number *N*	Turbine radius *R*[m]	The swept zone of turbine *A*_*S*_[m^2^]	Solidity *σ*	Blade chord length *C*[m]
NACA0021	3	0.515	1.5	0.25	0.085

**Fig 2:**
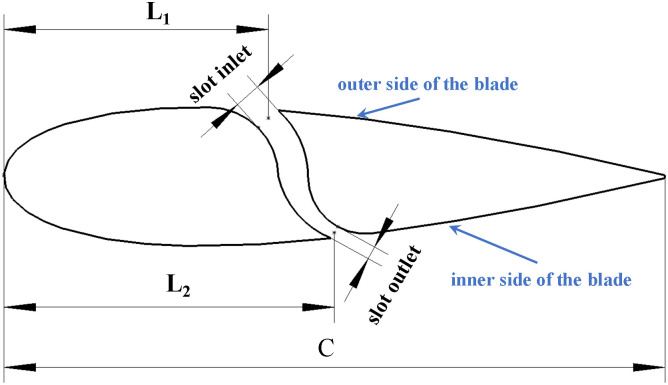
Geometric section of slotted airfoil.

The slotted airfoil adopts an S-shaped runner configuration, as shown in Fig 2. L1 and L2 denote the locations of the slot inlet and outlet, respectively. Although the reversal of flow direction at the slot inlet and outlet occurs when the blade operates under different wind regions, the design consistently locates the wider slot inlet on the outer side of the blade, while the narrower outlet is located on the inner side.

### 2.3. Computational domains and physical parameters

Accurate Prediction of VAWT performance using CFD simulation requires sufficiently small azimuthal increments (d*θ*) and grid sizes. The computational domain relative to the VAWT turbine diameter also needs to be sufficiently large for the wake to develop sufficiently and minimize the impact of blockage effects. Referring to the performance simulation prediction study of VAWT by Rezaeiha et al. [25] in 2017, after weighing the impacts of the computational volume and computational parameters, the computational domain of the model in this study was set to be 5*D* from the center of the turbine to the inlet of the computational domain, 10*D* to the outlet, and 10*D* in the width of the computational domain. Meanwhile, the time-step associated with the chosen azimuthal increment varies according to various turbine rotational speeds; both the time-step and maximum lattice size affect the stability parameter of the calculation. The stability parameter provides the criterion for assessing computational convergence and reproducibility, with values necessarily remaining below 0.3 throughout the simulation as mandated by technical protocols. Under the condition of sufficient calculation accuracy and a calculation stability parameter, the azimuthal increment was set to d*θ* ≤ 0.5°.

The air medium parameters were set according to the data measured in the experiments of Battisti et al. [24] that air temperature *T* = 25°C(298K), air density *ρ* = 1.155 kg/m^3^, turbulence intensity *I* = 1%, and dynamic viscosity *μ* = 1.834e-5 Pa·s.

### 2.4. Grid-independence validation and model validation

The LBM is frequently referred to as a meshless technique; however, it fundamentally relies on a lattice structure, such as a Cartesian grid, for simulation purposes. The spatial resolution (lattice density), particularly in regions near the blade wall and within the wake, plays a critical role in determining the accuracy of numerical computation results. Consequently, it is essential to independently validate the lattice refinement size for both the wall and wake zones. This validation should confirm discrepancy between computational results obtained from successive lattice refinements and those from the preceding lattice configuration converges to less than 2% to 5%. The validation of lattice independence for the Darrieus VAWT aims to assess its ability to capture DSV details at low TSR and total computation time. This validation was performed with a wind speed of V_∞_ of 16.18 m/s and TSR of 1.33, and was computed on a computer system equipped with a 12-thread Intel i5-12500 (3.0 GHz) processor.

**Fig 3:**
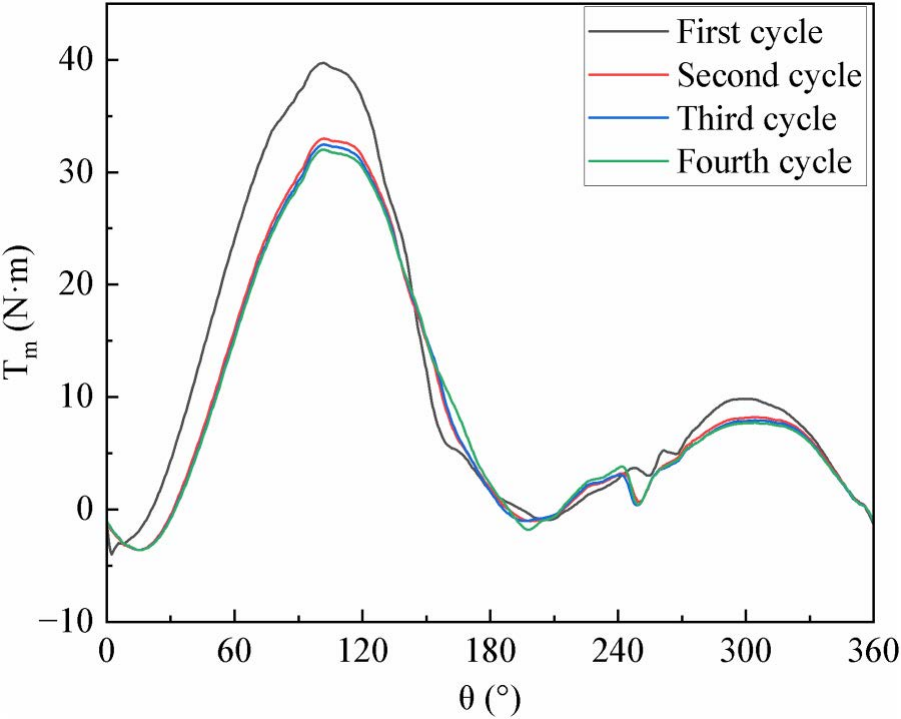
TSR = 1.33, 147 lattice numbers, the torque of blade during the baseline airfoil running cycle.

As shown in Fig 3, when the VAWT first starts operation, the blade wake is not sufficiently developed, resulting in a high peak torque of the blade in the first operation cycle. After two cycles, it starts to enter the stable cycle. Peak blade torque measured 32.024 N·m (Cycle 4) and 32.481 N·m (Cycle 3), with a marginal 1.43% inter-cycle deviation. This minimal variation, coupled with power curve convergence, verifies asymptotic stabilization of the flow field toward a steady state. To save the total calculation time of multiple cycles, the fourth cycle was selected as the calculation result in this study.

**Fig 4:**
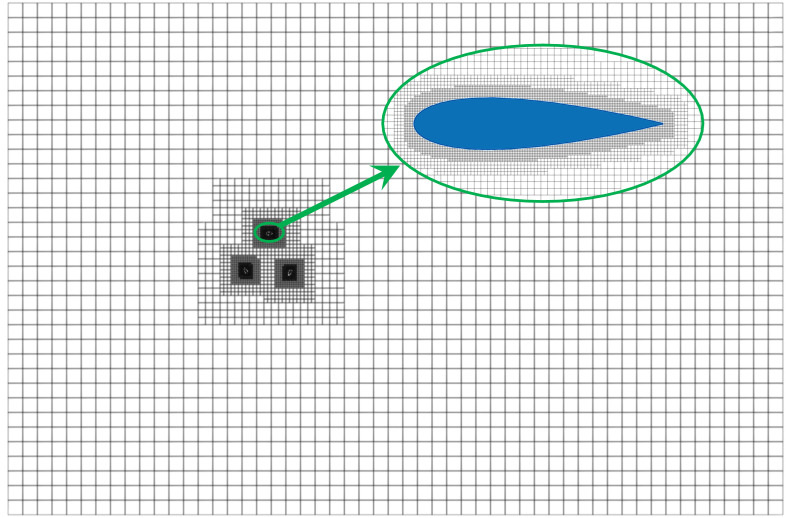
The encryption division results in computing the domain grid and grid around the blade.

The effect of lattice density on both the total computation time and the average power coefficient (*C*_*Pave*_) throughout a stabilization cycle is shown in Table 2. At a TSR of 1.33 and with the 167 distributed along the blade chord length, the calculation error rate of *C*_*Pave*_ compared to that obtained with 147 lattices is reduced to below 2%. This indicates that further refinement of the lattice resolution yields minimal improvements in the computational accuracy of the model. Additionally, at TSR of 0.5, the total computational time for the lattice number in 147 is as high as 155h owing to the generation of more vortices by the strong flow separation. Thus, to reduce the total computational time, the lattice size with a lattice number of 147 was selected. Fig 4 demonstrates the results of the computational domain meshing and meshing around the blade.

**Table 2:** Average power coefficient and total calculation time of three blades with various lattice densities.

TSR	Number of lattices on blade chord length	Global lattice size [m]	Wall and wake flow lattice size [m]	Three-blade average power coefficient (*C*_*Pave*_)	*C*_*Pave*_ error rate compared with the lattice numbers of the previous one	Total calculation time [h]
1.33	108	0.4	7.87037e-4	0.2521	—	15
1.33	127	0.3427	6.69336e-4	0.2969	17.76%	22
1.33	147	0.296	5.78125e-4	0.3156	6.3%	30
1.33	167	0.26	5.07812e-4	0.3212	1.77%	43
0.5	147	0.296	5.78125e-4	0.0821	—	155

**Fig 5:**
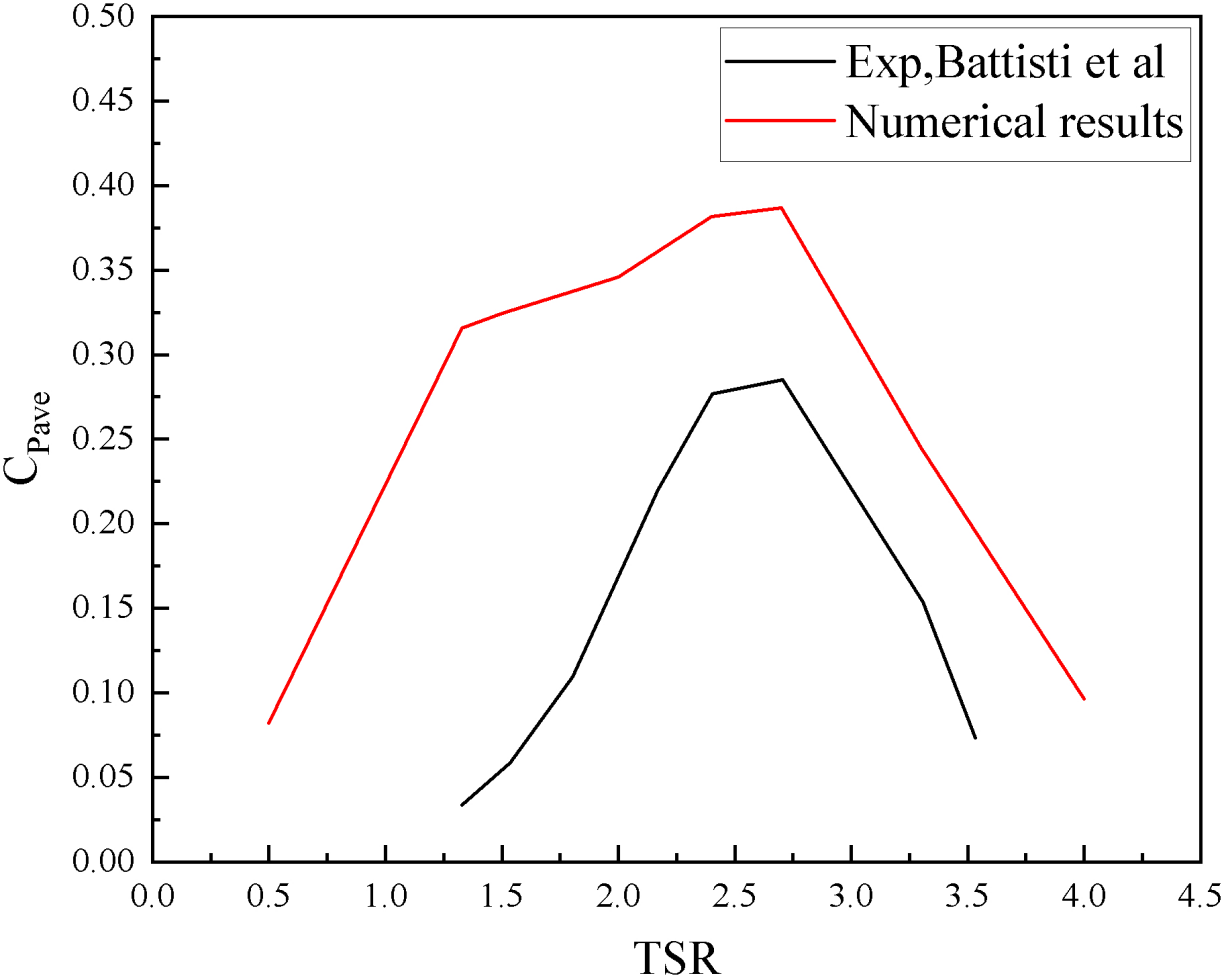
Comparison of numerical results with the experimental data of Battisti et al. [24].

The calculated results of *C*_*Pave*_ at various TSRs were compared with the experimental findings reported by Battisti et al. [24], as indicated in Fig 5, The directional trend and the peak point of the calculated results obtained from the 2D Darrieus VAWT model in this study were consistent with the experimental data.

When the Darrieus VAWT operates at low TSR, the disparity between the high-speed free stream and the blade rotational speed induces complex three-dimensional flow phenomena. These include the formation of tip vortices, trailing-edge vortices, and spanwise velocity dissipation, which substantially impair the blade performance. These 3D effects have a significant impact on the aerodynamic flow characteristics surrounding the VAWT [26]. The 2D simulation neglects the influence of these 3D effects, resulting in overestimated calculated values to varying extents [27]. Notably, the flow structure was substantially influenced by the tip vortices; the implementation of specialized tip designs can reduce the performance loss of blades induced by tip vortices [28,29]. Nonetheless, such analyses necessitate 3D simulations, which are beyond the scope of the present study. In 3D simulations, a large aspect ratio of the blade midplane can diminish the influence of tip vortices [30], thereby preserving characteristics that resemble those observed in 2D analyses. Some studies have equated 2D simulations to modeling blades of infinite height [29,31]. Consequently, as documented in prior research, 2D simulations retain efficacy in resolving fundamental aerodynamic mechanisms despite the inherent limitations in predicting 3D flow structures, while achieving computationally feasible solutions.

Concurrently, the present study model neglects the effects of eddy currents and flow velocity attenuation generated by the VAWT rotor shaft and connecting rod assemblies, which leads to the overestimation of calculated values [32,33]. However, owing to the substantial computational performance associated with the requirements of the LBM-LES methodology and the lack of previous reference cases, the potential impacts of the boundary conditions, smoothness of adaptive grid refinement, and the absence of surface roughness on the blade cannot be completely ruled out.

## 3. Results and discussion

In this study, we analyzed the impacts of the slot inlet and outlet locations on blade performance at TSRs of 0.5, 2.4, and 3.3 across different wind regions. The design parameters of the slot were not optimal. The purpose of this study was to observe how variations in slot location influence the flow field structure and the blade’s power coefficient, thereby providing insights to inform the design of the slot location parameters.

### 3.1. Effect of inlet location of the slots

The structural parameters of the slotted airfoil are listed in Table 3, with the outlet location, outlet width, and convergence ratio fixed, and the inlet location varied.

**Table 3:** Structural parameters of slotted airfoils with various inlet locations.

Inlet location *L*_*1*_[m]	Outlet location *L*_*2*_[m]	Outlet width[m]	Convergence ratio (inlet/outlet)	Inlet width[m]	Blade chord length *C*[m]	Configuration number
–	–	–	–	–	0.085	BL
0.1C	0.5C	0.02C	2	0.04C	0.085	0.1C-0.5C_2–2_SA
0.2C	0.5C	0.02C	2	0.04C	0.085	0.2C-0.5C_2–2_SA
0.3C	0.5C	0.02C	2	0.04C	0.085	0.3C-0.5C_2–2_SA
0.4C	0.5C	0.02C	2	0.04C	0.085	0.4C-0.5C_2–2_SA
0.5C	0.5C	0.02C	2	0.04C	0.085	0.5C-0.5C_2–2_SA

#### 3.1.1. TSR = 0.5.

**Fig 6:**
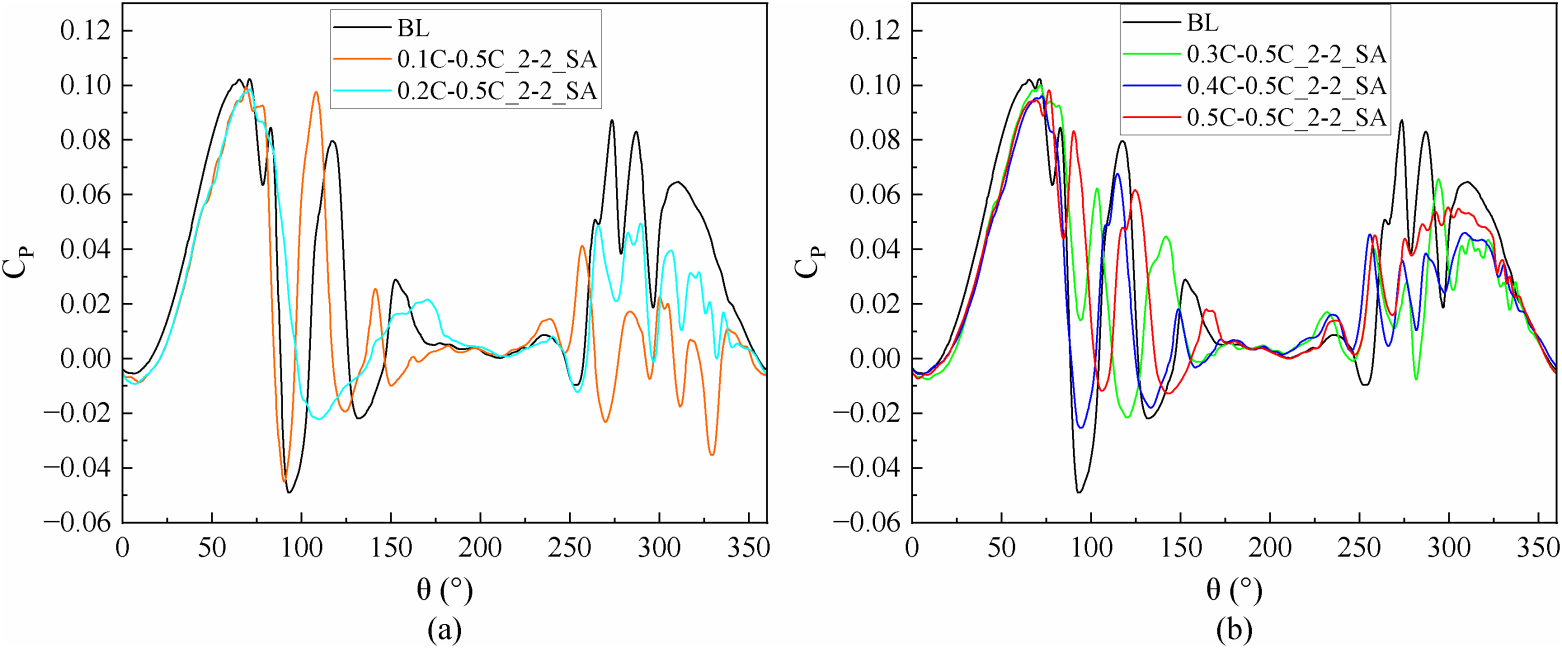
TSR = 0.5, instantaneous power coefficient of a slotted airfoil with various inlet locations versus baseline airfoil. (a) Slotted locations at 0.1C and 0.2C. (b) Slotted locations at 0.3C to 0.5C.

The instantaneous power coefficients (*C*_*P*_*)* of slotted airfoils with various inlet locations compared to the baseline airfoils are shown in Fig 6 during VAWT operation with a TSR of 0.5.

**Fig 7:**
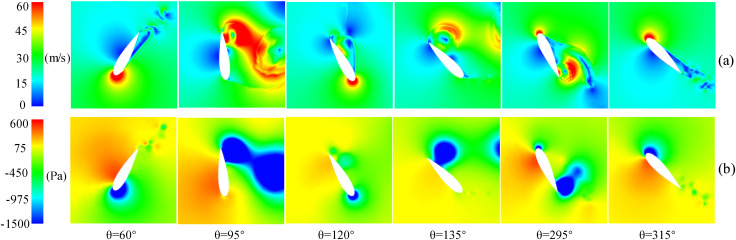
TSR = 0.5, the flow field of a baseline airfoil at various azimuthal angles. (a) Relative velocity contour. (b) Static pressure contour.

At 90° ≤ *θ* ≤ 180°, the upwind region and leeward region (90° ≤ *θ* ≤ 270°) overlap. In this overlapping region, the blades move in the direction of the incoming flow, and the AOA of the blades is large, which will facilitate flow separation near the trailing-edge. Therefore, two low peaks in the power coefficient caused by large-scale flow separation can be observed in Fig 6. The flow phenomenon can be observed in Fig 7, which demonstrates that the flow separation progressively develops from the trailing-edge to the leading-edge when the baseline airfoil is operated in the upwind region, covering the entire suction surface near 95° and 135°. This progression led to the elimination of the negative pressure near the leading-edge on the suction side, the loss of the lift force generated by the blade, and the formation of a large DSV at the trailing-edge. The presence of DSV induces considerable adsorption drag, ultimately causing the power coefficient of blade to become negative.

**Fig 8:**
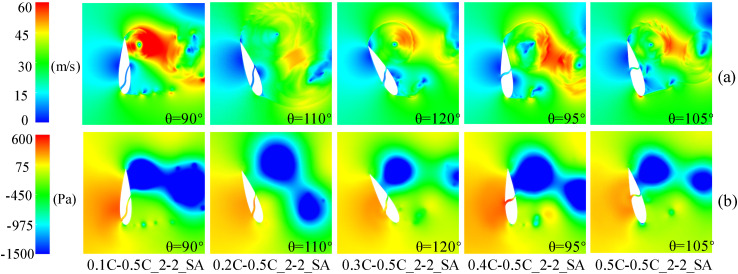
TSR = 0.5, azimuth of the worst stall for slotted airfoils with various inlet locations. (a) Relative velocity contour. (b) Static pressure contour.

In the upwind region, Fig 7 demonstrates a negative pressure distribution on the pressure side of the baseline airfoil, occurring prior to 0.2C from the leading-edge. Thus, as shown in Fig 8, it is difficult to form a high-speed jet when the slot inlet is arranged before 0.2C, which cannot weaken the DSV and attenuate the flow separation. Conversely, only the configuration with slot inlets and outlets located at 0.5C can effectively reduce the extent of flow separation and partially restore the negative pressure near the leading-edge. This slotted airfoil configuration achieved a 7.76% improvement in *C*_*Pave*_ relative to the baseline airfoil within the upwind region.

When the blade is running in the downwind region, Fig 7 demonstrates that although the baseline airfoil forms a DSV at the trailing-edge at *θ* = 295°, the extent of flow separation zone does not completely cover the entire suction side. Notably, the severity of flow separation in the downwind region was comparatively less pronounced than that observed in the upwind region.

**Fig 9:**
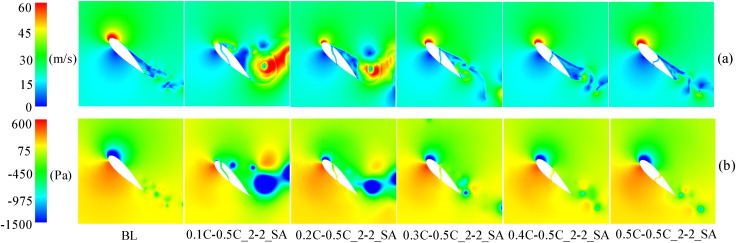
TSR = 0.5, baseline airfoils and slotted airfoils with various inlet locations at azimuth θ = 315°. (a) Relative velocity contour. (b) Static pressure contour.

Meanwhile, Fig 9 demonstrates that, in the downwind region, the reversal of the slot inlet and outlet causes the jets to flow in the opposite direction. Consequently, the jet outflowing from the slot inlet interferes with the flow that originally flowed near the airfoil profile, leading to the destruction of the flow structure at the leading-edge of the suction side. This disturbance prevents the formation of negative pressure near the slot inlet and results in an expanded flow separation zone, thereby increasing the possibility of DSV formation. Noteworthy, exhibited negative pressure predominantly concentrated before 0.5C on the suction side, whereas the slotted airfoil with the slot inlet location before 0.5C severely disrupted the primary negative pressure zone at the leading-edge of the blade. Therefore, as shown in Fig 6, in the downwind region, the power coefficients of the slotted airfoils with various inlet locations are smaller than those of the baseline airfoils and are more prone to fluctuations.

#### 3.1.2. TSR = 2.4, 3.3.

**Fig 10:**
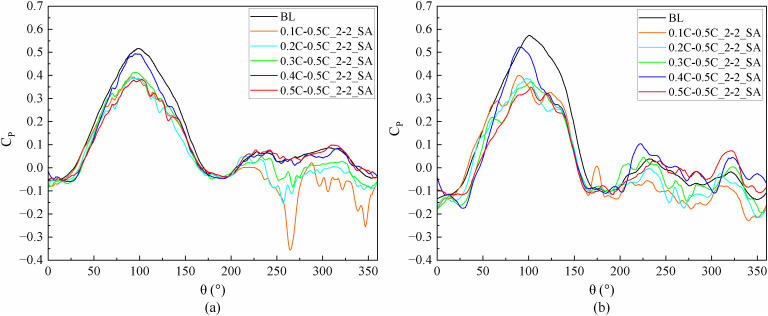
Instantaneous power coefficients of slotted airfoils with various inlet locations versus baseline airfoils. (a)TSR = 2.4. (b)TSR = 3.3.

At medium and high TSR (2.4, 3.3), the phenomena of flow separation and DSV are attenuated as the rotational speed of the blades rises; the jet flow within the slots is simultaneously affected by both the pressure distribution along the blades and the interactions between the fluid and solid surfaces. As illustrated in Fig 10, the power curves for each slotted airfoil exhibit similar patterns at TSR values of 2.4 and 3.3.

**Fig 11:**
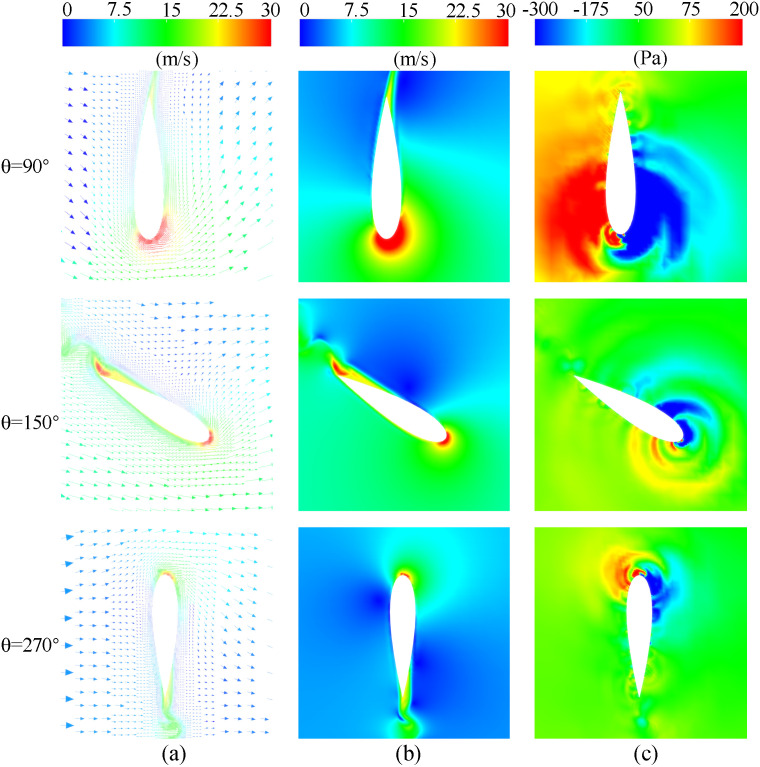
TSR = 2.4, the flow field of a baseline airfoil. (a) Fluid velocity vector plot. (b) Relative velocity contour. (c) Static pressure contour.

**Fig 12:**
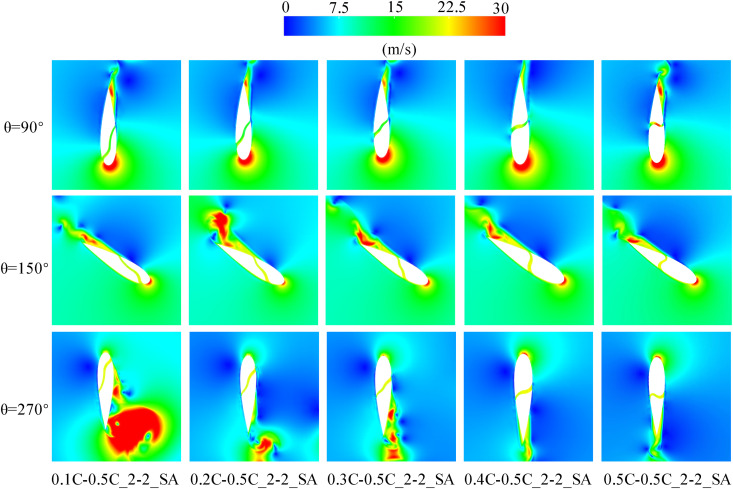
TSR = 2.4, relative velocity contours of slotted airfoils with various inlet locations.

A TSR of 2.4 was selected as a representative case for analysis, as illustrated in Fig 11. At *θ* = 90° within the upwind region, the thicker leading-edge of the blades squeezed the airflow due to the faster blade running velocity, resulting in a localized zone of increased airflow velocity and pressure on the pressure side of the baseline airfoil before 0.4C. after Downstream of 0.5C on the pressure side, the incoming flow was obstructed by the blades, forming a blocked low-velocity stagnation zone. When the slot inlet is positioned within either of these two zones, the airflow can more easily enter the slots and generate high-speed jets. Fig 12 demonstrates that the high-velocity jet, inclined at an angle, forms a blocking effect similar to an air wall, causing the airflow after the slot outlet to deviate from the blade surface and thereby exacerbating flow separation near the trailing-edge of the blade. The fluid within the separation zone stagnates on the suction side while moving rapidly with the blade, manifesting as an apparent high-speed wake at the blade’s trailing-edge. For the blade, carrying the separated fluid along with its movement necessitates additional power consumption. Fig 10 demonstrates that, in the upwind region, the slotted airfoil configuration with the slot inlet located at 0.4C exhibits a markedly higher *C*_*P*_ curve compared to other slotted airfoils. This improvement is attributed to the inlet’s strategic placement, which avoids the aforementioned high-velocity and stagnation airflow zone, thereby minimizing flow separation intensity and reducing blade drag forces.

In addition, Fig 12 demonstrates that at *θ *= 150° in the upwind region, the presence of the slot jet induces an enlargement of the flow separation region for all the slotted airfoils, culminating in the formation of a DSV. This enhanced flow separation consequently increases drag on the blades, thereby reducing the power coefficient of each slotted airfoil relative to the baseline airfoil throughout 90° ≤ *θ* ≤ 180°, as evident in Fig 10.

When the blade operates in the downwind region, the primary negative pressure zone on the suction side diminishes with increasing TSR. Fig 11 reveals that at *θ* = 270° in the downwind region, the negative pressure on the suction side concentrates predominantly within before 0.3C for the baseline airfoil. Correspondingly, Fig 12 demonstrates that when the slot inlet is located before 0.3C, the slot structure perturbs the leading-edge flow, triggering extensive flow separation and DSV formation, thereby elevating the blade’s drag forces.

**Fig 13:**
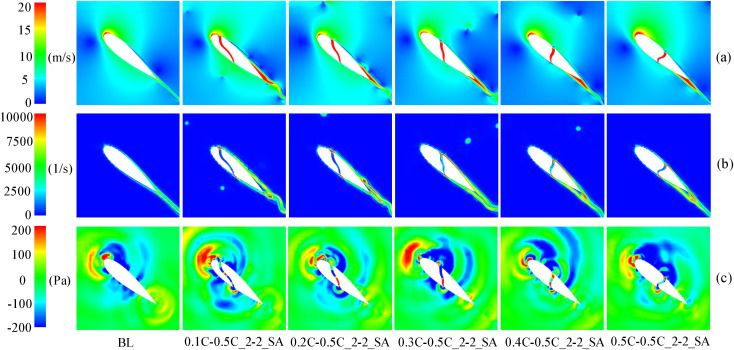
TSR = 3.3, slotted airfoils with various inlet positions and baseline airfoils. (a) Relative velocity contours. (b) Vorticity contours. (c) Static pressure contours.

Furthermore, Fig 10 indicates that at TSR of 2.4, the average power coefficient *C*_*Pave*_ of the 0.5C-inlet slotted airfoil exhibits a 16.24% enhancement relative to the baseline airfoil during the downwind operation. AT TSR of 3.3, the 0.4C-inlet configuration achieves a 63.62% improvement in *C*_*Pave*_ while the 0.5C-inlet variant yields a 43.28% increase. This phenomenon is illustrated in Fig 13. As depicted in Fig 13a, b, the slotted airfoils configuration with slot inlets located at 0.4C and 0.5C effectively replenishes the kinetic energy of the fluid on suction side, maintaining attached flow despite the turbulence and flow separation induced by the slot structure and outflow. Moreover, Fig 13a demonstrates that both slotted airfoils ingest air from the pressure side of the blade, resulting in a reduction of flow speed on this side. This speed reduction diminishes the negative pressure distribution on the pressure side (as evidenced in Fig 13c), thereby increasing the pressure differential across the blade surfaces and consequently enhancing the lift force.

### 3.2 Effect of outlet location of the slots

The structural parameters of the slotted airfoil are listed in Table 4, with the inlet location, outlet width, and convergence ratio fixed, and the outlet location varied.

**Table 4:** Structural parameters of slotted airfoils with various outlet locations.

Inlet location L_1_[m]	Outlet location L_2_[m]	Outlet width [m]	Convergence ratio (inlet/outlet)	Inlet width[m]	Blade chord length C[m]	Configuration number
–	–	–	–	–	0.085	BL
0.5C	0.6C	0.02C	2	0.04C	0.085	0.5C-0.6C_2–2_SA
0.5C	0.7C	0.02C	2	0.04C	0.085	0.5C-0.7C_2–2_SA
0.5C	0.8C	0.02C	2	0.04C	0.085	0.5C-0.8C_2–2_SA
0.5C	0.9C	0.02C	2	0.04C	0.085	0.5C-0.9C_2–2_SA

#### 3.2.1. TSR = 0.5.

**Fig 14:**
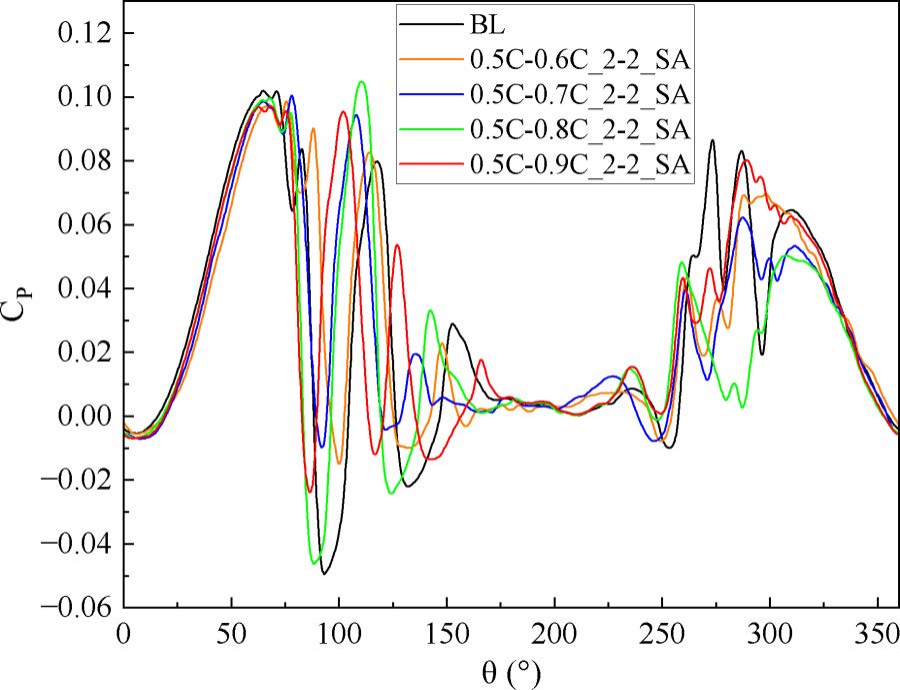
TSR = 0.5, instantaneous power coefficients of slotted airfoils with various outlet locations versus baseline airfoils.

The VAWT operation with TSR of 0.5, the instantaneous power of the baseline airfoil, and slotted airfoils with various outlet locations are shown in Fig 14.

**Fig 15:**
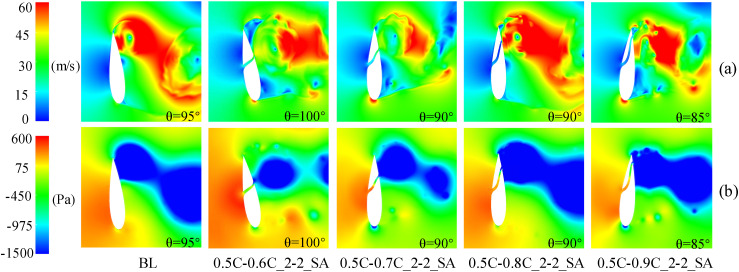
TSR = 0.5, baseline airfoils and slotted airfoils with various outlet locations at the azimuth of the worst stall. (a) Relative velocity contour. (b) Static pressure contour.

When the blade was running in the upwind region, Fig 14 demonstrates that the slotted configurations with the outlets at 0.6C and 0.7C achieve the *C*_*Pave*_ enhancements of 9% and 13.38%, respectively, relative to the baseline. At TSR of 0.5, the 0.5C-0.7C_2–2_SA configuration yields a total *C*_*Pave*_ of 0.0843 for all three blades’ whole cycle, representing a 2.68% increase over the baseline airfoil’s 0.0821. The associated flow phenomenon is revealed in Fig 15, the core zone of the DSV after 0.7C near the trailing-edge. When the slot outlet is located upstream near this zone, elevating the higher inlet and outlet pressure difference generates high-energy jets that fully encompass the entire DSV, effectively weakening the flow separation and the DSV. Conversely, although slot outlets at 0.8C and 0.9C experience significant pressure difference, their location within the core zone of the DSV prevents complete jet coverage, thus failing to mitigate flow separation effectively.

**Fig 16:**
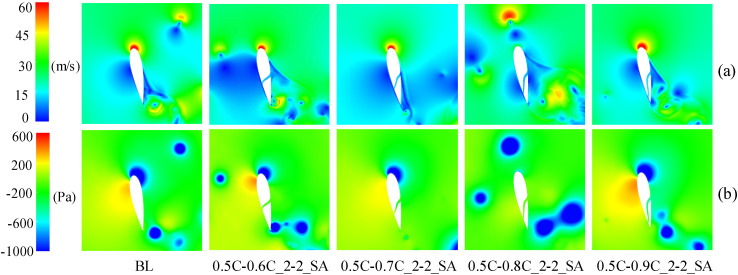
TSR = 0.5, slotted airfoils with various outlet locations and baseline airfoils have an azimuth θ = 280°. (a) Relative Velocity contour. (b) Static pressure contour.

When the blade operates in the downwind region, as shown in Fig 16, due to the small pressure difference at the trailing-edge of the blade, as the slot outlet approaches the trailing-edge, it causes a reduction in slot flow rate, thereby lessening the interference caused by the slotted airfoil to the negative pressure zone at the leading-edge. It is worth noting that due to the influence of the large DSV shed in the upwind region, the flow structure at the leading-edge of the slotted airfoil with an outlet location of 0.8C was disrupted. As a result, an obvious power coefficient decrease can be observed in Fig 14, which recovers when the blade leaves the influence zone of the DSV.

#### 3.2.2. TSR = 2.4, 3.3.

**Fig 17:**
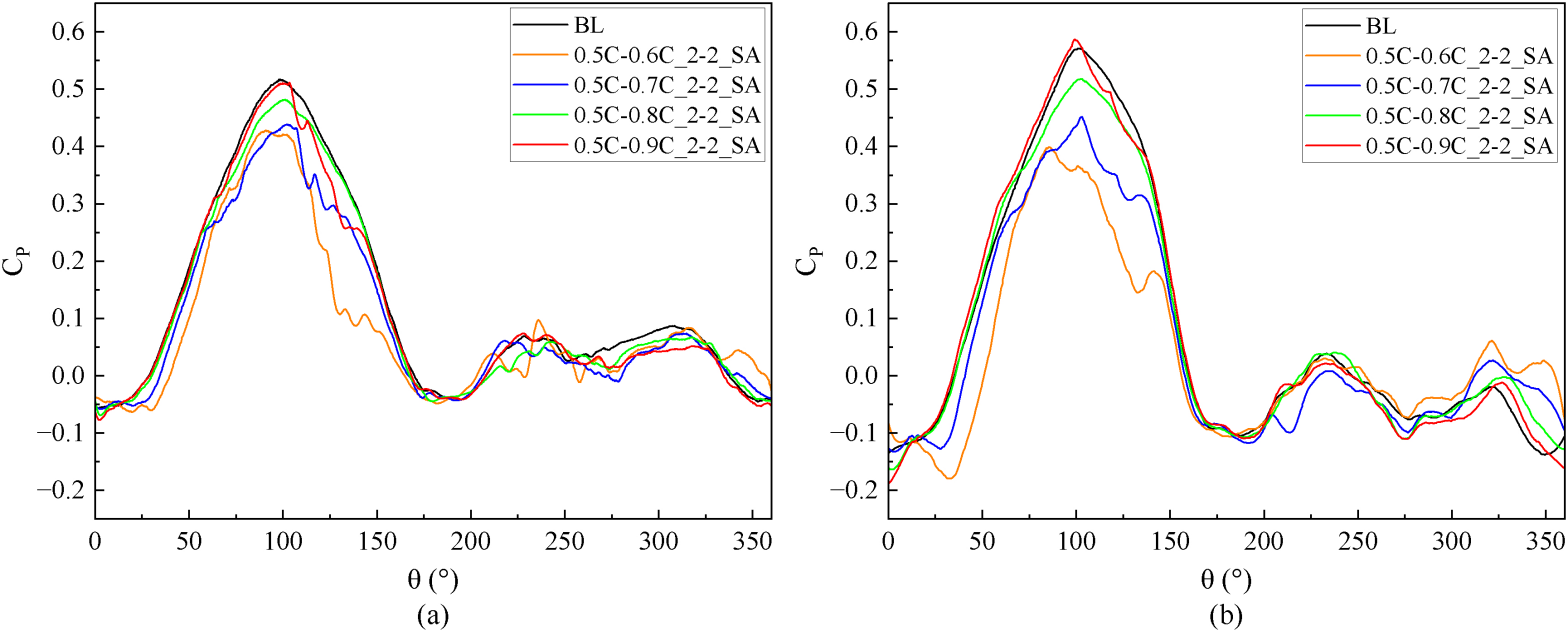
Instantaneous power coefficients of slotted airfoils with various outlet locations versus baseline airfoils. (a) TSR = 2.4. (b) TSR = 3.3.

**Fig 18:**
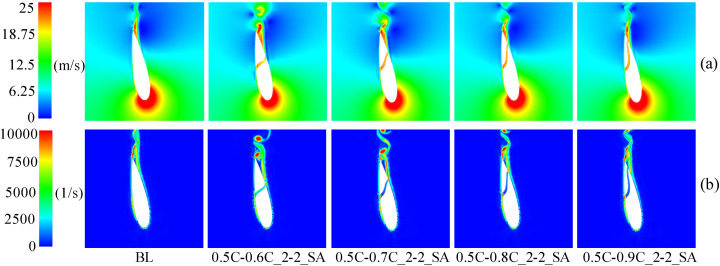
TSR = 2.4, slotted airfoils with various outlet locations and baseline airfoils. (a) Relative velocity contours. (b) Vorticity contours.

At medium and high TSR (2.4, 3.3), Fig 17 demonstrates the *C*_*P*_ of slotted airfoils with varying outlet positions. Obviously, within the upwind region, the *C*_*P*_ values of the slotted airfoils progressively converge to baseline levels as the outlet location approaches the trailing-edge. Notably, configurations with outlets at 0.9C exhibit curves closely resembling the baseline airfoils. This trend is further illustrated in Fig 18 at TSR of 2.4 and *θ* = 100°: outlets nearer trailing-edge reduce the flow separation extent. Specifically, a 0.9C-outlet location coincides with the original flow separation zone of baseline airfoil, exerting minimal disruption to the flow structure on suction side while generating a low drag force.

**Fig 19:**
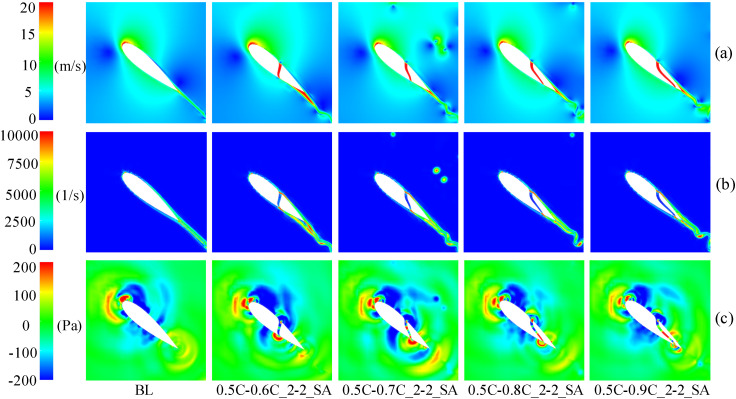
TSR  = 3.3, baseline airfoil and slotted airfoils with various outlet positions. (a) Relative velocity contours. (b) Vorticity contours. (c) Static pressure contours.

When the blade operates in the downwind region, Fig 17 indicates that at TSR of 3.3, the *C*_*Pave*_ exhibits enhancements of 62.27%, 7.25%, and 8.75% for slot outlet locations at 0.6C, 0.7C, and 0.8C, respectively. As depicted in Fig 19a, b, at *θ* = 320°, the presence of the slot disrupts the blade boundary layer and induces flow separation. Nevertheless, the configuration airflow with 0.6C-outlet effectively replenishes the kinetic energy on suction side, mitigating flow separation. Concurrently, Fig 19a demonstrates fluid deceleration on the pressure side, which diminishes the negative pressure distribution (as shown in Fig 19c) and thereby enhances the blade’s lift force. In contrast, the outlet of the other slotted airfoils is positioned near the trailing-edge, where the slot’s air intake is limited. This insufficiency fails to mitigate flow separation on the suction side caused by the slot structure, leading to increased drag forces of the blade.

## 4. Conclusion

Blade slots, as a PFC technique, necessitate a comprehensive investigation into how slot positioning and structural parameters influence the flow field characteristics surrounding the blade. This study utilizes a 2D numerical simulation of a Darrieus VAWT using the LBM-LES to examine the impact of varying slot location parameters of slotted airfoils on the performance of slotted airfoils operating in different wind regions. The principal findings are summarized as follows:

1. The implementation of slotted airfoils mitigates DSV formation at low TSR. However, at medium and high TSR, where blade flow separation is inherently reduced, the slot inlet near leading-edge adversely disrupts the blade’s original flow structure, inducing power loss. This observation corroborated with outcomes reported in prior research.
2. When the blade functions within the upwind region, attenuating the flow separation enhances the blade’s power coefficient. Specifically, at a low TSR of 0.5, the slotted airfoil configuration with the slot inlet located at 0.5C and the outlet at 0.7C yields a 13.38% increase in the *C*_*Pave*_ in the upwind region. Conversely, at moderate and high TSR of 2.4 and 3.3, the jet emanating from the slot outlet projects at an inclined angle, which disrupts the suction side flow, exacerbates the flow separation, and consequently increases blade drag forces.
3. For blades operating in the downwind region, it is critical to avoid slot-induced disturbances in the primary negative pressure zone near the leading-edge on the suction side. At a low TSR of 0.5, the slot inlet should be positioned at 0.5C or further downstream on the airfoil’s outer surface. At a medium TSR of 2.4, the slot configuration with both inlet and outlet locations at 0.5C enhances the *C*_*Pave*_ by 16.24% relative to the baseline. At a high TSR of 3.3, the two distinct configurations exhibit significant gains: the 0.4C-inlet/0.5C-outlet configuration achieves a 63.62% increase in *C*_*Pave*_; and the 0.5C-inlet/0.6C-outlet yields a 62.27% increase in *C*_*Pave*_. These improvements are attributed to the slot outflow’s ability to suppress small-scale flow separation on the suction side and reduce the negative pressure on the pressure side, thereby augmenting the pressure differential across the blade surfaces and enhancing the lift force, despite the turbulence and flow separation induced by the slot structure located near the blade mid-chord.

It is important to note that this study did not analyze the optimal configurations of other slot parameters, and further research is necessary to mitigate the adverse impacts of slotted airfoils at medium to high TSR. Additionally, it should be acknowledged that a single PFC technique is often inadequate to fully address the complex flow structure dynamics of Darrieus VAWT across varying operational conditions. Nonetheless, integrating multiple flow control strategies to develop a specialized blade design for Darrieus VAWT holds promise for achieving a high-efficiency blade configuration that comprehensively improves the flow field structure around the blade. Our considerations regarding the combined PFC approach are as follows:

a. The Genie flap is regarded as a particularly suitable PFC method to pair with a slotted airfoil, given that it is installed at the trailing-edge and its control mechanism does not conflict with the operational principles of the slotted airfoil.
b. The cavity structure, which functions as a vortex storage mechanism, can be analyzed in detail using the LES method to elucidate the intricate flow structures. Nevertheless, the spatial placement of the cavity may interfere with the slot.
c. Vortex generators, characterized by their 3D array structure, have the potential to suppress flow separation at medium to high TSRs, thereby potentially offsetting the limitations of the slotted airfoil, though their evaluation necessitates 2.5D or fully 3D simulations.
d. Furthermore, incorporating a one-way valve or an active switching mechanism within the slot could enable selective opening under specific TSR or wind regions, but at the expense of increased structural complexity and cost.

### Nomenclature

**Table:** 

*C*	Blade chord length [m]
*N*	Blade number [-]
*H*	Blade height [m]
*R*	Turbine radius [m]
*D*	Turbine diameter [m]
*A*_*S*_	The swept zone of turbine *A*_*S*_=2*HR*[m^2^]
*T*_*m*_	Torque [N·m]
*V*_*∞*_	Freestream velocity [m/s]
*ρ*	Air density [kg/m^3^]
*μ*	Dynamic viscosity [Pa·s]
*n*	Rotating velocity of turbine [rpm]
*V*_*S*_	Tip velocity of turbine *V*_*S*_ = 2π*Rn*/60 [m/s]
TSR	Tip speed ratio TSR = *V*_*S*_/*V*_*∞*_[-]
*θ*	Azimuthal angle [°]
AOA	Angle of Attack AOA = tan^-1^[sin*θ*/(TSR + sin*θ*)] [-]
*C*_*T*_	Torque coefficient ${C_{T}=T}_{m}/\left(\text{1}/\text{2}\rho V_{\infty }^{\text{2}}A_{S}R\right)$ [-]
*C*_*P*_	Power coefficient *C*_*P*_ = *C*_*T*_·TSR [-]
*C*_*Pave*_	Average power coefficient during one cycle [-]
*σ*	Solidity *σ *= *NC*/*D* [-]
*T*	Freestream temperature [°C]
*I*	Freestream turbulence intensity [-]
