# Supplementary material for: A study on the effect of slotted airfoil on the performance of Darrieus vertical axis wind turbines in different wind regions
Source: PLoS One. 2025 Oct 7;20(10):e0334110. doi: 10.1371/journal.pone.0334110 (PMC12503311; doi:10.1371/journal.pone.0334110)
Supplement: Attachment — Submitted filename: Response to Reviewers.docx [file pone.0334110.s001.docx]

Dear Hassan Elahi (Academic Editor) and Reviewers,

We sincerely appreciate the constructive feedback provided on our manuscript "	A study on the effect of slotted airfoil on the performance of Darrieus vertical axis wind turbines in different wind regions" (ID: PONE-D-25-02456). All editorial requirements and reviewer comments have been meticulously addressed in this revision. The key modifications include:

- Compliance with PLOS ONE formatting and data policies;

- Comprehensive language refinement and structural optimization;

- Implemented rigorous validation protocols for VAWT computational fluid dynamics (CFD);

- Enhanced methodological transparency through open-data practices.

Below, we provide point-by-point responses to journal requirements and reviewer comments. Responses in red font represent the authors' revisions. All referenced line numbers (e.g., L175) correspond to the "Revised Manuscript with Track Changes" file (*.docx format) with ALL markup displayed (Review > Show Markup > All Markup), and callouts provide revision notes.

**Response to Journal Requirements (Items 1-4):**

1. Please ensure that your manuscript meets PLOS ONE's style requirements, including those for file naming. The PLOS ONE style templates can be found at

<https://journals.plos.org/plosone/s/file?id=wjVg/PLOSOne_formatting_sample_main_body.pdf> and

<https://journals.plos.org/plosone/s/file?id=ba62/PLOSOne_formatting_sample_title_authors_affiliations.pdf>

We confirm that the manuscript has been reformatted according to PLOS ONE style templates, and the format of the figures was transformed using the NAAS tool. Should any non-compliance remain, we will promptly revise as requested. Thank you for your guidance.

2. Please note that PLOS ONE has specific guidelines on code sharing for submissions in which author-generated code underpins the findings in the manuscript. In these cases, we expect all author-generated code to be made available without restrictions upon publication of the work.

Please review our guidelines at https://journals.plos.org/plosone/s/materials-and-software-sharing#loc-sharing-code and ensure that your code is shared in a way that follows best practice and facilitates reproducibility and reuse.

This study did not involve author-generated code. All simulation configurations (boundary conditions, solver parameters, mesh settings) are integrated into an executable .xfp file (Xflow 2022). The associated blade model (.nfb) and parametric CAD files (.SLDPRT, .STEP) are packaged in the same Zenodo deposit [DOI: <https://doi.org/10.5281/zenodo.16736530>], enabling one-click simulation replication.

3. We note that your Data Availability Statement is currently as follows:

“All relevant data are within the manuscript and its Supporting Information files.”

Please confirm at this time whether or not your submission contains all raw data required to replicate the results of your study. Authors must share the “minimal data set” for their submission. PLOS defines the minimal data set to consist of the data required to replicate all study findings reported in the article, as well as related metadata and methods (https://journals.plos.org/plosone/s/data-availability#loc-minimal-data-set-definition).

For example, authors should submit the following data:

- The values behind the means, standard deviations and other measures reported;

- The values used to build graphs;

- The points extracted from images for analysis.

Authors do not need to submit their entire data set if only a portion of the data was used in the reported study.

If your submission does not contain these data, please either upload them as Supporting Information files or deposit them to a stable, public repository and provide us with the relevant URLs, DOIs, or accession numbers. For a list of recommended repositories, please see <https://journals.plos.org/plosone/s/recommended-repositories> .

If there are ethical or legal restrictions on sharing a de-identified data set, please explain them in detail (e.g., data contain potentially sensitive information, data are owned by a third-party organization, etc.) and who has imposed them (e.g., an ethics committee). Please also provide contact information for a data access committee, ethics committee, or other institutional body to which data requests may be sent. If data are owned by a third party, please indicate how others may request data access.

The minimal data set required to replicate our findings has been deposited in Zenodo:

**Computational Models**:

- ` VAWT Assembly .SLDPRT` (SOLIDWORKS 2022 parametric blade)

- ` Blade Model .STEP` (Neutral CAD format)

**Simulation Files**:

- ` Main Simulation .xfp` (Xflow 2022 simulation file with integrated settings)

- ` Blade Model .nfb` (Xflow-generated model)

**Raw Results**:

- ` Blade’s Torque and Calculate Stability .txt` (Real-time parameters)

- ` Raw Power Coefficient .xlsx` (Raw calculation data that has not been smoothed)

**Access URL**: <https://doi.org/10.5281/zenodo.16736530>

4. When completing the data availability statement of the submission form, you indicated that you will make your data available on acceptance. We strongly recommend all authors decide on a data sharing plan before acceptance, as the process can be lengthy and hold up publication timelines. Please note that, though access restrictions are acceptable now, your entire data will need to be made freely accessible if your manuscript is accepted for publication. This policy applies to all data except where public deposition would breach compliance with the protocol approved by your research ethics board. If you are unable to adhere to our open data policy, please kindly revise your statement to explain your reasoning and we will seek the editor's input on an exemption. Please be assured that, once you have provided your new statement, the assessment of your exemption will not hold up the peer review process.

Related data are now permanently accessible via Zenodo. This includes raw computational outputs and metadata necessary for reproducibility.

**Response to Reviewer Comments (#1, #2, #3):**

**Reviewer #1**: The main title is develops very well. The main idea is develops and discusses by chronological idea very well. The English is well written. The manuscript is discusses STEM very well. It is such a great idea when it is discussing in physics model for the study.

We sincerely appreciate the Reviewer's positive assessment of our work.

**Reviewer #2**: Please improve the quality of the english that has been used in the writing. It is quite repetitive and somewhat hard to read.

Can the analysis extend upto a 3D simulation? Could you add that?

The result section should be easy to read. It is too redundant.

We thank the reviewers for their suggestions.

**Improve English quality:**

The manuscript underwent comprehensive language refinement using professional enhancement tools (Grammarly, Paperpal, QuillBot), ensuring strict preservation of original technical content and structural integrity while optimizing grammatical precision and lexical sophistication.

**Limitations of 3D Simulation:**

Regrettably, although 3D LES can provide more details, its computational cost far exceeds the available resources. We discussed the rationality of 2D simulation of VAWT in the manuscript and cited previous literature. Prior studies validate that 2D simulations effectively capture key Darrieus VAWT aerodynamics. (L187-207 of chapter 2.4)

**Reduction of Redundancy:**

Unnecessary content was deleted:

- Section 3.1.1: L252;

- Section 3.1.2: L321 and L326;

- Section 3.2.1: L413;

- Section 4: L475 and L485.

The relevant content of the main findings was discussed in more detail:

- Section 3.1.2: L334 and L350;

- Section 3.2.2: L443-457.

**Reviewer #3**: Several important issues should be addressed to improve the quality and clarity of the manuscript:

Major Comments:

• The title accurately reflects the scope of the study and is appropriate for the research conducted.

We appreciate the reviewers' positive comments on the consistency between the title of the manuscript and the scope of the study.

• The abstract is informative but too dense; it should be more concise and clearly separated into motivation, method, and key findings.

This was a very constructive comment, and we revised the abstract to clarify the research question and to describe the main findings. (Abstract: L22-44).

• The study addresses a relevant problem in VAWT performance and applies a credible simulation approach (LBM + LES), which is a strong point.

We thank the reviewers for their recognition of the LBM-LES methodology used in our study.

• The literature review provides sufficient background, but citation style is inconsistent and many references are grouped (e.g., [13–18]) without clear attribution of specific points.

We recognize this issue, and disaggregated grouped citations [13-18] (Section 1: L57-78) and [23-25] (Section 2.1: L104-106) with specific attributions.

The reference [6] was replaced to improve the reference relevance (References: L579), and the original reference [14] (References: L605) with insufficient relevance was deleted.

• Authors should consider enriching the literature review and strengthening citation relevance by including the following recent studies:

 https://doi.org/10.3390/wind5010004

 https://doi.org/10.3390/en18071601

 https://doi.org/10.1371/journal.pone.0305745

 https://doi.org/10.3390/su17104654

We are grateful for the suggested references. After searching, the subjects of these four papers are:

- Optimization of wind turbine control system ([https://doi.org/10.3390/wind5010004]）

- Optimal Protection Coordination for Microgrid ([https://doi.org/10.3390/en18071601])

- Optimal planning and partitioning of multi-microgrid ([https://doi.org/10.1371/journal.pone.0305745])

- Wind Power Cybersecurity ([https://doi.org/10.3390/su17104654])

However, our study addresses VAWT Blade aerodynamic flow control. Differences in paper topics may be controversial, and we will prioritize citations if the topic of future research is relevant to the topic of the recommended paper.

• The novelty of the work is implied but not explicitly stated; it would benefit from a clearer articulation of how this study improves upon or differs from previous ones.

Benefiting from the reviewers' suggestions, we took note of this and highlighted the novelty of the study in the abstract and conclusion (Abstract: L35-42; Section 4: L503-508).

• The numerical setup is well detailed, and grid-independence is performed, but error metrics like RMSE or percentage deviation from experimental data are missing.

This was our mistake. In the aerodynamic simulation of vertical-axis wind turbines, an increase in the number of lattice causes the average power coefficient to converge to a certain value when verifying grid-independence. We expressed the simulation indicator of “error convergence” of the average power coefficient as “growth rate,” which caused ambiguity. We made corrections and provided explanations (Section 2.4: L146 and L165; Section 2.4: L175 in Table 2).

• There are broken references and unresolved figure/table labels (e.g., “Error! Reference source not found”) which must be fixed.

The broken Table 2 was fixed (Section 2.4: L165), and the all cross-references were verified again before submitting the manuscript.

• The simulations are 2D, yet the authors themselves acknowledge the importance of 3D effects in DSV formation; this limitation should be discussed more transparently and critically.

Compared with 3D models, two-dimensional turbulence models are indeed limited in terms of simulation accuracy. We refer to previous studies on this limitation and discuss why “the 2D model of Darrieus VAWT can also reflect general flow field characteristics” (Section 2.4: L187-207).

• Results for different slot inlet/outlet locations and TSRs are insightful and match expected aerodynamic behavior, but the discussion sometimes remains too qualitative.

We have revised the description method in “3. Results and Discussion”. The analysis will observe and interpret the flow phenomena in the figures so that the discussion corresponds to the phenomena in the figures and avoiding being overly qualitative. (Starting from Section 3.1.1: L227)

• Figures are often unclear, lack units, or have low resolution, making interpretation difficult. Improved labeling and explanations are necessary.

All figures were generated at the journal's maximum resolution specification (**600 DPI**) to ensure clarity during magnification. To enhance interpretability, font sizes in textual annotations were systematically increased.

For power curve visualizations, minimal auxiliary markings were intentionally preserved to maintain visual clarity while facilitating trend identification across complex airfoil datasets.

Additionally, an inaccuracy in Fig 1 was rectified: the angle of attack descriptor "AOA" was replaced with the Azimuthal angle symbol θ (theta) in accordance with conventional aerodynamic notation. (Section 2.2: L114)

• Quantitative comparisons should include uncertainty bounds or confidence intervals to support performance claims.

We acknowledge the importance of statistical uncertainty quantification for computational studies. However, given the extreme computational demands of our LBM-LES approach (requiring 15–155 hours per simulation case under current resource constraints, as documented in Table 2, Section 2.4: L175), performing repeated runs to generate traditional confidence intervals (e.g., 95% CI) was pragmatically unfeasible within the revision timeline.

To ensure rigorous data reliability, we implemented a two-tiered validation protocol:

**Convergence-Based Validation:**

- All simulations strictly adhered to Xflow’s stability criterion (Stability Parameter < 0.3), This industry-standard metric guarantees solution convergence and reproducibility. We emphasized this metric in the paper (Section 2.3: L132).

**Methodological Safeguards:**

- flow field time steady-state processing (Section 2.4, L157)

- Grid-independence Validation (Section 2.4: L165)

- Validation against experimental profiles trend (Section 2.4: L183 in Fig 5)

While classical uncertainty bounds remain desirable, our approach aligns with CFD studies for VAWT, such as reference [15] and [16] (References: L613 and L616), where single-converged-run protocols are accepted when supported by:

- Strict convergence metrics;

- Grid-independence;

- Related experimental benchmarking.

We also corrected the input errors in the manuscript when we collated the data (Section 2.4: L175 in Table 2; Section 3.1.2: L350). The raw data, including Stability Parameter and meta-file, have been uploaded to the Zenodo permanent database for easy access by readers. Access URL: https://doi.org/10.5281/zenodo.16736530

• The conclusion reiterates key points well, but uses vague terms (“significantly improves”, “slight flow separation”) without consistent numerical backing.

Ambiguous and imprecise terminology has been systematically eliminated from the manuscript, with flow phenomena now described directly and performance enhancements substantiated by simulation data. (Abstract: L38; Section 3.1.2: L335 and L343; Section 4: L479 and L494)

• The statement about combining multiple PFC strategies is interesting but undeveloped; the authors should briefly suggest specific complementary techniques.

In the conclusion at the end of the article, we propose some PFC methods that may be suitable for combination with slotted wing types, and we hope that this will be useful for future research directions (Section 4: L518).

• Reference formatting is inconsistent; some DOIs are separated onto new lines and some sources are outdated or not peer-reviewed.

Considering scholars' concerns about the sources of the literature, we removed academic papers that had not undergone peer review: [15] (References: L608). Updated the outdated NASA literature sources: [8] and [9] (References: L588 and L592).

Reference citations were reformatted to comply with journal template specifications, transitioning from superscript to standard numerical notation (Sections 1 and 2). Additionally, hyphenation parameters were optimized throughout the document to ensure all DOIs maintain co-linear alignment with their respective reference entries.

• Language throughout the paper needs editing by a native or fluent English speaker due to frequent grammar issues and awkward phrasing.

Comprehensive linguistic refinement was implemented via specialized software (Grammarly, QuillBot), enhancing grammatical coherence and terminological precision while simultaneously ensuring uncompromised preservation of original technical content and structural integrity.

• Overall, the methodology is solid and the problem is well motivated, but the manuscript requires substantial revision in presentation, clarity, and depth of analysis.

We express sincere gratitude to the reviewers for their insightful suggestions, which have been instrumental in enhancing the manuscript's rigor. In Section 3 (Results and Discussion), redundant content was removed (Section 3.1.1: L252; Section 3.1.2: L321 and L326; Section 3.2.1: L413) with strengthened figure-text integration implemented.

Supplemental figures were added (Section 3.1.2: L361; Section 3.2.2: L461) to enable detailed analysis of pivotal findings (Section 3.1.2: L350; Section 3.2.2: L443).

• Recommendation: Major revision. The technical content is promising, but presentation and language issues must be addressed, and more rigorous analysis (quantitative and comparative) is needed.

We sincerely appreciate the reviewers' acknowledgment of our work. Notwithstanding the extended research duration and challenges in acquiring datasets for robust statistical analysis, rigorous model validation was conducted—including grid-independence validations—to ensure full reproducibility. All analytical results derive exclusively from raw data, employing no methodologies that compromise academic integrity.

Additionally, while preserving all technical content intact, professional language enhancement tools were utilized to refine linguistic expression in the initial manuscript draft.

Sincerely,

Liqi Luo (on behalf of all authors)

Submission Contact & First Author

Guilin University of Electronic Technology

Email: [2217128852@qq.com](mailto:2217128852@qq.com)
